# Can Implicit or Explicit Time Processing Impact Numerical Representation? Evidence From a Dual Task Paradigm

**DOI:** 10.3389/fpsyg.2019.02882

**Published:** 2020-01-08

**Authors:** Maria Grazia Di Bono, Caterina Dapor, Simone Cutini, Konstantinos Priftis

**Affiliations:** ^1^Department of General Psychology, University of Padua, Padua, Italy; ^2^Department of Neuroscience, Biomedicine and Movement Sciences, University of Verona, Verona, Italy; ^3^Department of Developmental Psychology and Socialization, University of Padua, Padua, Italy

**Keywords:** number representation, time representation, number-time interactions, ATOM, visuospatial attention, dual task

## Abstract

Whether the human brain processes various types of magnitude, such as numbers and time, through a shared representation or whether there are different representations for each type of magnitude is still debated. Here, we investigated two aspects of number-time interaction: the effects of implicit and explicit processing of time on numbers and the bi-directional interaction between time and number processing. Thirty-two participants were randomly assigned into two experimental groups that performed, respectively, a Single task (number comparison, with implicit time processing) and a Dual task (number comparison as a primary task, with explicit time processing as a secondary task). Results showed that participants, only in the Dual task, were faster and more accurate when processing large numbers paired with long rather than short durations, whereas the opposite pattern was not evident for small numbers. Moreover, participants were more accurate when judging long durations after having processed large rather than small numbers, whereas the opposite pattern emerged for short durations. We propose that number processing influences time processing more than vice versa, suggesting that numbers and time might be at least partially independently represented. This finding can pave the way for investigating the hierarchical representation of space, numbers, and time.

## Introduction

Processing temporal and numerical quantities (e.g., the number of objects in a set and their size; the time elapsing before action onset) is essential for representing the external world and for preparing suitable motor programs for action. The issue of whether the human brain processes different types of magnitude (e.g., numbers and time) through a shared representation or whether there are different magnitude representations, has been the focus of recent behavioral and neuroimaging studies (for review, see Bueti and Walsh, 2009; Bonato et al., 2012).

At the behavioral level, studies investigating the mental representation of numbers, by means of number comparison or parity judgment tasks, have revealed three fundamental effects (Moyer and Landauer, 1967; Dehaene et al., 1993): the distance effect (i.e., faster processing for larger numerical differences in a number pair); the size effect (i.e., faster processing of smaller numbers); and the Spatial-Numerical Association of Response Codes (SNARC) effect (i.e., faster left/right-sided responses to small/large numbers, respectively). While the distance and the size effects reveal the presence of an analogical format, similar to that for other perceptual magnitudes, the SNARC effect points to the existence of a spatially oriented mental number line (MNL) where numerically smaller numbers are represented on the left of larger numbers (at least in Western cultures). As for numbers, converging evidence has shown that also temporal durations can be processed through a spatial representation. The STEARC (Spatial TEmporal Association of Response Code) effect, originally reported by Ishihara et al. (2008), showed that temporal durations are represented along a Mental Time Line (MTL), where short durations are on the left of the longer ones (for review, see Bonato et al., 2012).

Whether the SNARC (or the STEARC) effect is a demonstration of the spatial organization of numbers (or time), has been questioned by Proctor and Cho (2006), who suggested that these effects could also be explained in terms of match/mismatch of polarity correspondence between numbers and the response space. According to Proctor and Cho, number magnitude (and by extension temporal magnitude) and the response space are coded with a polarity that can be negative (e.g., associated with the left space and small numbers) or positive (e.g., associated with the right space and large numbers). This account, however, cannot explain empirical evidence such as the distance or the size effects. Furthermore, the studies of Stoianov et al. (2008) and Di Bono et al. (2012), where non-spatially lateralized responses were used (i.e., vocal responses without polarity), have shown that visuospatial cues can influence the processing of numerical and temporal information, respectively. Moreover, Di Bono et al. (2012) have reported the presence of a distance-like effect for temporal durations, which mirrors the distance effect in the numerical domain. These studies have provided evidence of early number-space and time-space interactions, even before response selection (i.e., at the level of mental representations).

Whether these spatial representations for numbers and time are distinct or partially overlapping, remains an open question. A reference theoretical framework is the “A Theory Of Magnitude” (ATOM) proposed by Walsh (2003); see also Bueti and Walsh (2009). According to this theory, number, time, and space processing stem from the same core magnitude processing system. At the neural level, it is currently debated whether numerosity is processed by dedicated neural mechanisms, or whether it is a part of a common magnitude system which processes every quantity-related information. Similarly, the existence of a dedicated system for time perception remains an open question (for review, see Dormal and Pesenti, 2012). Some studies have shown that temporal processing relies on dedicated neural networks (for reviews, see Ivry and Schlerf, 2008; Grondin, 2010). In contrast, other studies have shown that time is strongly associated with numerosity, as a part of a generalized magnitude system (Lourenco and Longo, 2010; de Hevia et al., 2014).

A further number of studies has supported the existence of shared processing mechanisms among numerosity, time, and space (Hubbard et al., 2005; Srinivasan and Carey, 2010; Cai and Connell, 2015; Schwiedrzik et al., 2016). Moreover, several studies have reported a close association between numbers and time (Meck and Church, 1983; Dormal and Pesenti, 2012; Dormal et al., 2012). Nevertheless, neuropsychological studies (e.g., Cappelletti et al., 2009) and Transcranial Magnetic Stimulation (TMS) studies (e.g., Dormal et al., 2008) have shown a dissociation between the two domains. A marked asymmetry between the two domains emerged in dual task paradigms: numbers seem to influence time more than vice-versa (Dormal et al., 2006, 2012; Bueti and Walsh, 2009; Cappelletti et al., 2009; Winter et al., 2015). Furthermore, cross-dimensional adaptation paradigms (Tsouli et al., 2019a) have revealed that duration adaptation affects numerosity estimation. In contrast, there is insufficient evidence to support/reject the effect of numerosity adaptation on duration judgment. Successively, Tsouli et al. (2019b), investigated how duration influences numerosity perception, and they showed that at least two temporal mechanisms are involved. Tsouli et al. (2019b) found that: (i) the effect of “adaptation to duration” on numerosity perception is independent from adaptation duration (channel based hypothesis); (ii) the effect of “adaptation to numerosity” on numerosity perception is driven by the total duration of the adaptation trials (strength-of-adaptation hypothesis).

In the studies of Tsouli et al. (2019a, b), the unidirectional influence of time on numerosity perception has been conceptualized according to the ATOM theory in terms of a Bayesian framework (Martin et al., 2017). Martin et al. (2017) suggested that when sensory information for numerosity processing accumulates over a variable (short/long) duration, the uncertainty in temporal processing adds noise to the accumulation of sensory information for numerosity, altering its perception.

Most of the evidence supporting ATOM comes, however, from neuroimaging studies, which have shown that both numerosity and duration processing rely on spatially overlapping cortical networks (Dormal et al., 2012; Hayashi et al., 2013; Skagerlund et al., 2016). Nevertheless, such overlapping pattern of activity does not necessarily imply a shared mechanism because it might have different origins. Indeed, the poor temporal resolution of functional Magnetic Resonance Imaging (fMRI) does not allow for an analysis of the temporal dynamics of the brain. Moreover, the severe constraints of the conventional fMRI data analysis technique (the General Linear Model – GLM) cannot disentangle different neural circuits located in the very same brain regions (Di Bono et al., 2017).

A different theoretical perspective, however, points to visuospatial attention as a possible mechanism mediating the activation of a (spatially based) layout for temporal and numerical representations (see Di Bono et al., 2012, in the temporal domain; Stoianov et al., 2008, in the numerical domain). Indeed, Di Bono et al. (2012) showed that task-irrelevant, lateralized visuospatial primes affect auditory duration judgments: responses to short (long) durations were faster when the auditory target was paired with left-(right-) sided rather than with right-(left-) sided primes. Moreover, Di Bono et al. showed that physical space influences time even when a lateralized, spatially encoded response is not required by the task, suggesting that space and time interact at the level of mental representations, instead at the level of response selection.

In the same vein, Stoianov et al. (2008) showed that visuospatial–numerical interactions do occur, even before response selection. In the temporal domain, the role of spatial attention in mediating the spatial representation of temporal durations has been suggested also by Marin et al. (2016), who have shown that left neglect, a spatial attention disorder in right-brain-damaged patients, affects the processing of the MTL, especially in its leftward portion, in a way that is proportional to the severity of the deficit. The role of visuospatial attention in mediating the activation of a spatial layout for representing both numbers and time, has been reported by Casarotti et al. (2007): numbers must be explicitly processed in order to elicit a shift of spatial attention, which in turn affects the perception of the temporal order of visual events. This finding suggests that: (i) the interaction among numbers and space is not symmetrical: space influences numbers more than vice-versa; (ii) the deployment of visuospatial attention could mediate the mental representation of both numbers and time.

According to the MNL hypothesis, there are long-term associations between individual numerical representations and the spatial codes. In contrast, the working memory account (van Dijck and Fias, 2011; van Dijck et al., 2014; Fias and van Dijck, 2016; Abrahamse et al., 2016) posits that numbers are linked to spatial codes as a function of their ordinal position within a sequence. In both cases, it is impossible to rule out the role of visuospatial attention in mediating these spatial effects. Analogously for temporal quantities, Starr and Brannon (2016) have shown that visuospatial (and not verbal) working memory influences the interaction between space and time, suggesting that the interference between spatial and temporal representations are due to processing constraints in visuospatial working memory. Moreover, Cai et al. (2018) showed that cross-dimensional (space-time) interaction arises as a result of memory interference, when spatial length and duration information co-exist in working memory. Specifically, the extension and the direction of these interactions depend on the relative memory noise of the target and the interfering dimension. Cai et al. (2018) interpreted their findings in terms of a Bayesian model in which the estimation of a magnitude is based on the integration between the noisily encoded target magnitude and the prior knowledge about the magnitudes that co-vary across dimensions (i.e., space and time).

The general aim of the present study was to investigate the effects of implicit and explicit processing of time on numbers, before the response selection (i.e., at the level of mental representations). Our hypotheses were that, similarly to space-number (Stoianov et al., 2008; Kramer et al., 2011) and space-time interactions (Di Bono et al., 2012), number-time interaction could emerge at the level of mental representations, prior to response selection. Furthermore, in contrast to Fischer et al. (2003), a series of studies on number-space interactions (Fischer et al., 2003; Galfano et al., 2006; Casarotti et al., 2007; Fattorini et al., 2015), have shown that perceiving numbers does not automatically cause shifts of spatial attention, unless numbers are explicitly processed. Thus, as in the case of space-number interactions, it is challenging to investigate whether only explicit processing of temporal duration should interfere with number processing. Moreover, we aimed to investigate the assumptions of ATOM in terms of clear testable predictions: if the representations of numbers and time overlap, we would find a reciprocal interference (with similar characteristics) across the two domains, both in RTs and accuracies. In contrast, the presence of asymmetrical interactions should provide evidence for, at least, partially distinct representations of time and space.

The specific aim of the present study was, thus, twofold: (1) to investigate the effects of implicit and explicit processing of time on numbers; (2) to investigate the bi-directional interaction between time and numbers. We employed a priming paradigm, adapted from the ones used in Stoianov et al. (2008) and Di Bono et al. (2012), using verbal instead of spatial responses, to rule out the hypothesis that possible interactions take place at the level of response selection. Moreover, to manipulate the implicit/explicit processing of time (e.g., Casarotti et al., 2007 in number-space interaction), we employed a dual task paradigm. In a mixed experimental design, two groups of participants performed, respectively, a single task (implicit time processing; task1: number comparison, after just perceiving temporal durations) and a dual task (explicit time processing; task 1: number comparison; task 2: temporal duration judgment). We manipulated both the cardinal and the ordinal dimension of time, by varying the duration of the temporal stimuli and their onset with respect to that of numbers.

According to ATOM (Walsh, 2003), we hypothesized that if the representation of durations overlapped with that of numbers, we should observe that: (i) the time-number congruency effect is symmetrical (i.e., the influence of time on numbers is similar to that of numbers on time); (ii) this symmetrical congruency effect is automatic (i.e., time influence numbers both when durations are merely perceived in the Single Task, and when they are explicitly processed in the Dual Task). Alternatively, according to the MNL/MTL hypothesis (e.g., Stoianov et al., 2008; Di Bono et al., 2012) or the working memory account (e.g., van Dijck et al., 2014; Starr and Brannon, 2016), distinct representations for numbers and time could exists, and spatial attention could be the common mechanism underlying them. In this perspective, we expected an asymmetrical time-number interaction, and only when time was explicitly processed. Indeed, distinct spatial representations for durations and numbers could exist and could interact only when explicitly required by specific tasks.

## Materials and Methods

### Participants

Thirty-two students of the University of Padua (15 females; mean age: 23.2, *SD* = 1.36; 30 right-handed, one left-handed, and one ambidextrous) volunteered to take part in the study. The optimal number of participants (i.e., 30) was estimated using the software G^∗^POWER 3 (Faul et al., 2007), according to the experimental design, and by using the following parameters: alpha level = 0.01, Power = 0.95, effect size = 0.25. We recruited 32, instead of 30 participants, to manage for the presence of possible outliers. None of the participants had visual or auditory problems. Participants were divided into two groups, each one composed by sixteen participants. Each group took part only in one of the two Experiments (i.e., Single Task or Dual Task). All participants provided their informed consent to participate to the study. The study was approved by the Ethics Committee of the Department of General Psychology, University of Padua.

### Apparatus and Stimuli

An INTEL^®^ CORE^TM^ 2 computer was used, with Microsoft Windows XP Professional Operating System, version 2002, Service Pack 3; CPU: 6320 @ 1.86 GHz; RAM: 0.97 GB; Update frequency: 100 Hz; Colors: max 32 bits; Video card: Intel^®^ 6965 Express Chipset Family (chip type: Intel^®^ GMA 3000; NAC tip: Internal; memory size: 256 MB; Bios information: Internal video Bios); Sound Card: SoundMAX Integrated Digital HD Audio.

Visual stimuli, white on a black background screen, were generated using E-prime 2.0 (Psychology Software Tools, Inc., 2012) and were presented on a 21′′ NEC Multisync P1150 monitor (resolution: 1024 × 768). They consisted of a fixation cross (font: Arial; font size: 36; height: 1°; width: 1°) that was presented on the screen for 500 ms and an Arabic digit (i.e., integers in the range [2–4] and [6–8]; Font: Arial; font size: 52; maximum height: 2°; maximum width: 1.5 degree of visual angle) that was presented on the screen for 300 ms. The auditory primes, presented through two loudspeakers (Philips MMS110 Multimedia Speaker System), located on the monitor’s sides, consisted of 441-Hz sinusoidal tones (amplitude = 0.8), that could have a duration of 100 or 150 ms (short duration), and 300 or 350 ms (long duration). Participants’ vocal reaction times (RTs) were recorded by a microphone (ATR20 Audio-Technica Cardiod Low Impedance). To measure accuracy, participants’ responses were also recorded and coded online, by the experimenter, through a keyboard. The whole experiment was also audio-recorded by means of a Sony IC Recorder ICD-MX20, to double check offline for the responses’ accuracy.

### Procedure

Each participant was tested individually in a silent and dimly lit room. Before starting the experiment, participants were asked to read and sign the informed consent. Thereafter, participants were asked to place their head on a chinrest that was positioned at a distance of 57 cm from the monitor.

#### Single Task Condition

In the Single Task, each trial started with the fixation cross. After 500 ms, the cross disappeared and the Arabic digit was presented on the center of the screen for a duration of 300 ms. The onset of the auditory cue always preceded the onset of the Arabic digit (*forward* Stimulus Onset Asynchrony – SOA). For half of the trials, the onset of the target corresponded to the offset of the prime (no-overlap conditions), whereas for the other half of the trials, the two stimuli overlapped for a constant duration of 100 ms (overlap conditions). Specifically, in the overlap condition the onset of the Arabic digit was 100 ms before the offset of the prime. In the no-overlap conditions the SOA could last 100, 150, 300, or 350 ms, depending on the duration of the tone. In contrast, in the overlap conditions the SOA could last 0, 50, 200, or 250 ms, depending on the duration of the tone. During the inter-trial interval, a blank display of random duration (1 or 1.4 s) was presented.

While maintaining their eyes on central fixation, participants were asked to perform a number comparison task. As soon as they saw the target, they had to respond orally, by using two arbitrary non-words: half of the participants were instructed to say “Ti”/”To” if the target number was smaller/larger than 5. The response mapping was reversed (i.e., “To”/”Ti” if the target number was lesser/greater than 5) for the other half of the participants. We decided to use these two non-words, characterized by the same initial consonant, in order to control any timing bias in the activation of the voice-key for the verbal response. Both speed and accuracy were stressed in the instructions. Hence, in the Single Task condition, participants were not asked to perform the duration judgment task (*implicit* processing).

**FIGURE 1:**
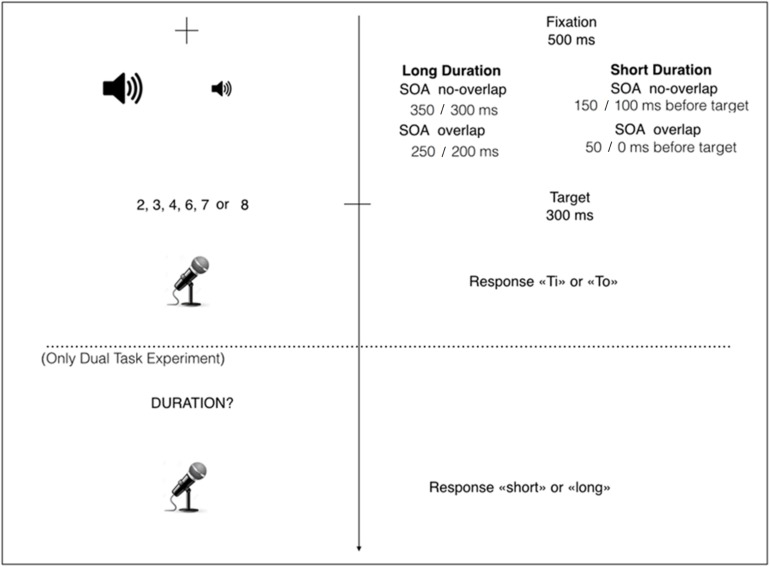
Stimuli presentation. Each trial started with the presentation of the fixation cross for 500 ms. Immediately after, the auditory cue was presented, that could be short (100 or 150 ms) or long (300 or 350 ms). The auditory cue could overlap with the target for 100 ms (overlap conditions) or not (no-overlap conditions). At 100 ms before the tone’s offset (overlap conditions) or at the same time as the tone’s offset (no-overlap conditions), the Arabic digit was presented for 300 ms. As soon as the Arabic digit appeared, participants had to answer, as quickly and accurately as possible, if the number was smaller or larger than 5, by saying the non-words “Ti” and “To” (response pattern counterbalanced between participants). After that, only the participants assigned to the Dual Task condition had to report if the tone was short or long, by saying the words “short” and “long,” at the appearance of the word “DURATION?”

All the participants received a practice session of 12 trials, followed by four blocks, each one composed of 144 experimental trials; between one block and the following block there was an 1-min break (Figure 1). For the entire experiment, the experimenter was seated behind the participants.

#### Dual Task Condition

The procedure of the Dual Task condition was identical to that of the Single Task, except that after having performed number comparison, participants were asked to perform also a duration judgment task (*explicit* processing): as soon as they saw the Italian word “DURATA?” (i.e., “DURATION?”), they had to judge, as quickly and accurately as possible, whether the duration of the presented tone was short or long, by using the Italian words “*breve*” and “*lunga*” (i.e., “short” and “long,” respectively).

### Experimental Design

We used a mixed experimental design. The between-participants independent variable was Task (two levels: Single vs. Dual). The within-participants independent variables were SOA (two levels: overlap vs. no-overlap), Duration (two levels: short vs. long), and Number (two levels: small vs. large). The dependent variables were RTs and accuracy.

## Results

We excluded three outlier participants, whose percentage of errors and omissions, in one of the two tasks (only in the numerical task for the Single Task condition), was above 2.5 *SD* from the mean. The cut-offs were 9.52% for the Single Task condition, and 38.07% (number comparison task) and 34.71% (duration judgment task) for the Dual Task condition. In particular, we excluded one participant from the Single Task condition (with 15.45% of errors and omissions in the numerical task) and two participants from the Dual Task condition (participant 1 with 48.96% of errors/omissions in the numerical task; participant 2 with 39.76% of errors/omissions in the temporal task). Thus, statistical analyses were performed on a total of 15 participants in the Single Task condition, and 14 participants in the Dual Task condition.

### RTs

Trials with errors and/or omissions, in at least one of the two tasks (number comparison and duration judgment), were excluded from the analysis: 1.45% of trials in the Single Task condition (1.23% errors and 0.24% omissions) and 14.25% in the Dual Task condition (2.9% errors and 5.8% omissions in the number comparison task, 7.55% errors in the duration judgment task). Then, for each participant and condition, we excluded RTs below and above 2.5 *SD* from the mean: the percentage of discarded trials was 2.76% in the Single Task condition and 2.05% in the Dual Task condition. We computed the mean RT for each participant in each condition of the number comparison task. Then, mean RTs were subjected to a four-way mixed ANOVA with SOA (no-overlap vs. overlap), Duration (short vs. long) and Number (small vs. large) as within-participants factors, and Task (single vs. dual) as the between-participants factor. We used *t*-tests for exploring the significant interactions. In particular, one-tailed *t*-tests were used for exploring interactions expected by *a priori* hypotheses, whereas two-tailed *t*-tests were used for exploring unexpected effects. Confidence intervals, reported in the figures, were computed by using the single method of O’Brien and Cousineau (2014).

The ANOVA (see Supplementary Table 1) revealed a main effect of SOA, *F*(1, 27) = 293.59, *p* < 0.001, $\eta_{p}^{2}$ = 0.92. Responses were faster in the case of no overlap between the two stimuli (duration and number; Single Task: *M* = 528.70 ms, *SEM* = 2.514; Dual Task: *M* = 838.82 ms, *SEM* = 3.808), with respect to the case of overlap (Single Task: *M* = 554.51 ms, *SEM* = 2.514; Dual Task: *M* = 891.07 ms, *SEM* = 3.808). There was also a main effect of Duration, *F*(1, 27) = 120.30, *p* < 0.001, $\eta_{p}^{2}$ = 0.82. Responses were faster with long durations (Single Task: *M* = 520.80 ms, *SEM* = 5.500; Dual Task: *M* = 837.27 ms, *SEM* = 6.952), than with short durations (Single Task: *M* = 562.41 ms, *SEM* = 5.500; Dual Task: *M* = 892.62 ms, *SEM* = 6.952). The effect of Task was also significant, *F*(1, 27) = 51.20, *p* < 0.001, $\eta_{p}^{2}$ = 0.65. RTs in the Single Task condition were faster (*M* = 541.61 ms, *SEM* = 21.66) than in the Dual Task condition (*M* = 864.94 ms, *SEM* = 42.39).

The interaction between SOA and Task was significant, *F*(1, 27) = 34.45, *p* < 0.001, $\eta_{p}^{2}$ = 0.56. Independent-samples *t*-tests showed that the simple effect of Task emerged in both the two SOA conditions^1^, even if it was more pronounced, as indexed by Cohen’s *d*, in the overlap condition, *t*(27) = −7.44, *p* < 0.001, *MD* = −336.6, *SEM* = 46.16, 95% CI*_*MD*_* [−∞, −259.5], *d* = 3.310, 95% CI*_*d*_* [1.391, 5.162], than in the no-overlap condition, *t*(27) = −6.85, *p* < 0.001, *MD* = −310.1, *SEM* = 46.22, 95% CI*_*MD*_* [−∞, −233.0], *d* = 2.546, 95% CI*_*d*_* [1.540, 3.527].

The interaction between SOA and Duration was also significant, *F*(1, 27) = 8.50, *p* = 0.007; $\eta_{p}^{2}$ = 0.24. Paired-samples *t*-tests showed that the effect of SOA emerged with both the long and short durations^2^, but, as indexed by the Cohen’s *d*, it was more pronounced with the short durations, *t*(27) = −14.26, *p* < 0.001, *MD* = −45.23, *SEM* = 3.173, 95% CI*_*MD*_* [−51.73, −38.73], *d* = 0.221, 95% CI*_*d*_* [0.156, 0.286], than with the long durations, *t*(28) = −6.48, *p* < 0.001, *MD* = −31.91, *SEM* = 4.929, 95% CI*_*MD*_* [−42.00, −21.82], *d* = 0.157, 95% CI*_*d*_* [0.093, 0.219]. Moreover, the simple effect of Duration emerged in both the SOA conditions^3^, but it was more pronounced, as indexed by the Cohen’s *d*, in the overlap, *t*(28) = 10.71, *p* < 0.001, *MD* = 54.90, *SEM* = 5.127, 95% CI*_*MD*_*[44.40, 65.41], *d* = −0.262, 95% CI*_*d*_*[−0.345, −0.178], than in the no-overlap condition, *t*(28) = 8.08, *p* < 0.001, *MD* = 41.59, *SEM* = 5.149, 95% CI*_*MD*_*[31.04, 52.14], *d* = −0.210, 95% CI*_*d*_* [−0.283, −0.134].

Crucially, the expected interaction between Number and Duration was significant, *F*(1, 27) = 4.82, *p* = 0.037, $\eta_{p}^{2}$ = 0.15. Paired-samples *t*-tests (one-tailed) showed a congruency effect for large numbers, *t*(28) = −8.34, *p* < 0.001, *MD* = −56.34, *SEM* = 6.754, 95% CI*_*MD*_* [−∞, −44.85], *d* = −0.271, 95% CI*_*d*_* [−0.365, −0.175]. Participants were faster in processing large numbers when they were preceded by long durations (*M* = 672.42 ms, *SEM* = 6.754), than by short durations (*M* = 728.76 ms, *SEM* = 6.754). Nevertheless, the congruency effect did not emerge for small numbers.

The three-way interaction SOA × Duration × Task was significant, *F*(1, 27) = 5.55; *p* = 0.026; $\eta_{p}^{2}$ = 0.17. Simple effects analyses showed that the two-way interaction SOA × Duration was significant in the Single Task, *F*(1, 14) = 22.99, *p* < 0.001, $\eta_{p}^{2}$ = 0.62, but not in the Dual Task (see Supplementary Tables 2, 3).

The expected tree-way interaction between Number, Duration, and Task was not significant, although the effect size was rather large, *F*(1, 27) = 3.66; *p* = 0.066; $\eta_{p}^{2}$ = 0.12. According to our hypotheses we expected to find a significant interaction between Number and Duration (i.e., the congruency effect), only for the Dual Task. Thus, we further explored this hypothesis by separating RTs for the Single and the Dual Task. Then, we conducted two Repeated Measures (RM) ANOVAs, one for each task.

None of the other factors or interactions was significant (all the *F*_*s*_ ≤ 2.58; see details in Supplementary Table 1).

#### Exploring the Interaction Number × Duration × Task

##### Single Task

As reported in Supplementary Table 2, the interaction between Number and Duration was not significant in the Single Task, *F*(1, 14) = 0.35; *p* = 0.564; η^2^*_*p*_* = 0.02.

##### Dual Task

**FIGURE 2:**
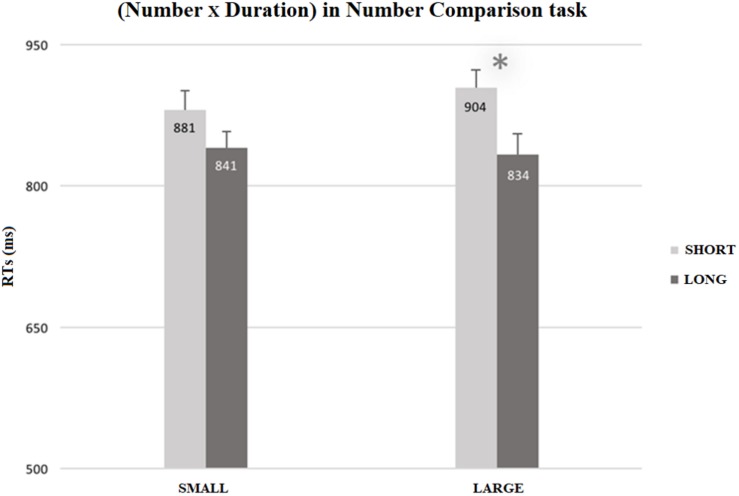
Mean RTs (ms) for the number comparison task in the Dual Task condition, as a function of Number (small vs. large) and Duration (short vs. long). RTs for large numbers were faster when they were preceded by long durations (*M* = 833.69 ms, *SEM* = 6.105) than by short ones (*M* = 904.44 ms, *SEM* = 5.503). The error bars indicate the confidence intervals (95%), corrected for within-participants designs (O’Brien and Cousineau, 2014; single method).

As reported in Supplementary Table 3, the interaction between Number and Duration was not significant, *F*(1, 13) = 4.46; *p* = 0.055; η^2^*_*p*_* = 0.25. Nevertheless, the effect size was very large. According to our specific *a priori* hypothesis, paired-samples *t*-tests (one-tailed), between the congruent (i.e., small numbers-short durations, large numbers-long durations) and incongruent (i.e., small numbers-long durations, large numbers-short durations) conditions, showed a congruency effect only for large numbers, *t*(13) = −6.16, *p* < 0.001, *MD* = −70.76, *SEM* = 11.48, 95% CI*_*MD*_* [−∞, −50.43], *d* = −0.439, 95% CI*_*d*_* [−0.654, −0.218]. Participants were faster when processing large numbers preceded by long durations (*M* = 833.69 ms, *SEM* = 11.479), rather than by short durations (*M* = 904.44 ms, *SEM* = 11.479). The congruency effect did not emerge for small numbers (see details in Supplementary Table 3 and Figure 2).

#### Summary: RTs

With reference to our hypotheses, we can state that the results on the RTs showed that congruency between number and duration processing was present only in the Dual Task, and only for large numbers. Thus, only when time was explicitly processed (i.e., in the Dual Task) and only when participants had to judge that the target number was larger than 5 (i.e., the reference number), we found that RTs, in the number comparison task, were modulated by the judgment of the temporal durations.

### Accuracy

For investigating the nature (symmetric or asymmetric) of the interaction between numbers and durations, we exploited the experimental dual task paradigm, and analyzed the participants’ accuracy in both the first task (i.e., number comparison) that was performed in both the Single and the Dual task condition, and the second task (i.e., duration judgment) that was performed only in the Dual task condition.

Trials with omissions in the Single or the Dual Task conditions were excluded from the analysis: we excluded 5.8% of trials in number comparison task and 0.88% of trials in duration judgment task, for a total of 6.51%. For both number comparison and duration judgment, we computed the mean accuracy percentage, for each participant in each condition. For the number comparison task, we subjected the mean accuracies to a four-way mixed ANOVA, using SOA (non-overlap vs. overlap), Duration (short vs. long) and Number (small vs. large) as within-participants factors, and Task (Single vs. Dual) as a between-participants factor. For the duration judgment task (performed only in the Dual task), we subjected the mean accuracies to a three-way RM-ANOVA using SOA, Duration and Number as within-participants factors. *T*-tests were used for following-up the significant interactions. Confidence intervals, reported in the figures, were calculated with the single method of O’Brien and Cousineau (2014).

#### Accuracy in Number Comparison

As reported in Supplementary Table 4) the Mixed ANOVA revealed a main effect of Duration [*F*(1, 27) = 10.09, *p* = 0.004, η ^2^*_*p*_* = 0.27]: participants were more accurate when numbers were paired with long rather than with short durations. The double interaction SOA × Task was also significant [*F*(1, 27) = 4.49, *p* = 0.043, η ^2^*_*p*_* = 0.14]: participants were less accurate in the Dual rather than the Single Task condition, only when numbers and durations overlapped. Moreover, we found a significant double interaction Duration × Task [*F*(1, 27) = 4.96, *p* = 0.035, η ^2^*_*p*_* = 0.16], explained by the fact that the effect of Duration was significant in the Dual task condition [*t*(13) = 3.14, p = 0.008; *M*_L__ONG_ = 0.978, *SEM*_LONG_ = 0.0086; *M*_SHORT_ = 0.97, *SEM*_SHORT_ = 0.0088], but not in the Single task [*t*(14) = 0.89, *p* = 0.39] condition.

Crucially, the interaction between Number and Duration was significant [*F*(1, 27) = 4.87, *p* = 0.036, η ^2^*_*p*_* = 0.15] and was explained by a congruency effect for large numbers [i.e., participants were more accurate when they processed large numbers paired with long rather than short durations, *t*(28) = 2.58, *p* = 0.015; *M*_LONG_ = 0.985, *SEM*_LONG_ = 0.005; *M*_SHORT_ = 0.973, *SEM*_SHORT_ = 0.006], whereas no significant congruency effect emerged for small numbers [*t*(28) = 0.79, *p* = 0.44]. Finally, the triple interaction Number × Duration × Task was significant [*F*(1, 27) = 4.24, *p* = 0.049, η ^2^*_*p*_* = 0.14]. To follow-up the triple interaction, we conducted two separate three-way RM-ANOVAs, one for each condition (Single and Dual).

##### Exploring the Interaction Number × Duration × Task

###### Single Task

In the Single task condition, no main effects nor interactions emerged (all *F*_*s*_ ≤ 2.22; see Supplementary Table 5).

###### Dual Task

**FIGURE 3:**
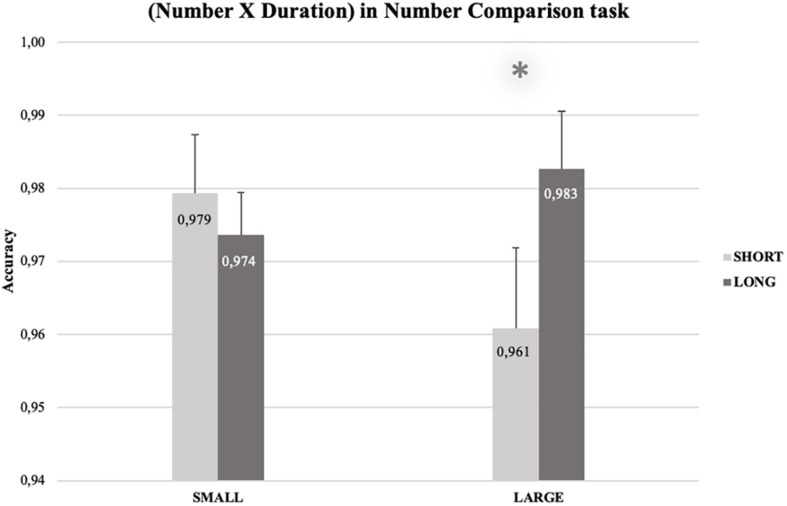
Percentage of correct answers for the number comparison task, as a function of Duration (short vs. long) and Number (small vs. large). Accuracy for numbers larger than 5 was higher when they were preceded by long durations (*M* = 0.983, *SEM* = 0.004) than by short ones (*M* = 0.961, *SEM* = 0.006). The error bars indicate confidence intervals (95%), corrected for within-participants designs (O’Brien and Cousineau, 2014; single method).

The RM-ANOVA for the Dual task condition (see Supplementary Table 6), revealed a main effect of Duration [*F*(1, 13) = 9.79, *p* = 0.008, η ^2^*_*p*_* = 0.429]. Accuracy was higher for long durations (*M* = 0.978; *SEM* = 0.002), than for the short ones (*M* = 0.970; *SEM* = 0.002). The interaction between Number and Duration was significant [*F*(1, 13) = 5.88, *p* = 0.031, η ^2^*_*p*_* = 0.311]. Paired-samples *t*-tests (one-tailed) showed a congruency effect for large numbers, *t*(13) = 2.92, *p* = 0.006, *MD* = 0.022, *SEM* = 0.007, 95% CI*_*MD*_* [0.009, ∞], *d* = 0.559, 95%, CI*_*d*_* [0.120, 0.982]: accuracy for large numbers was higher when they were preceded by long (*M* = 0.983, *SEM* = 0.007), rather than by short (*M* = 0.961, *SEM* = 0.007) durations. In accordance with the RTs’ results, the congruency effect did not emerge for small numbers (Figure 3). No other main effects or interaction were significant [all the *F*_*s*_(1, 13) ≤ 2.84].

#### Accuracy in Duration Judgment

**FIGURE 4:**
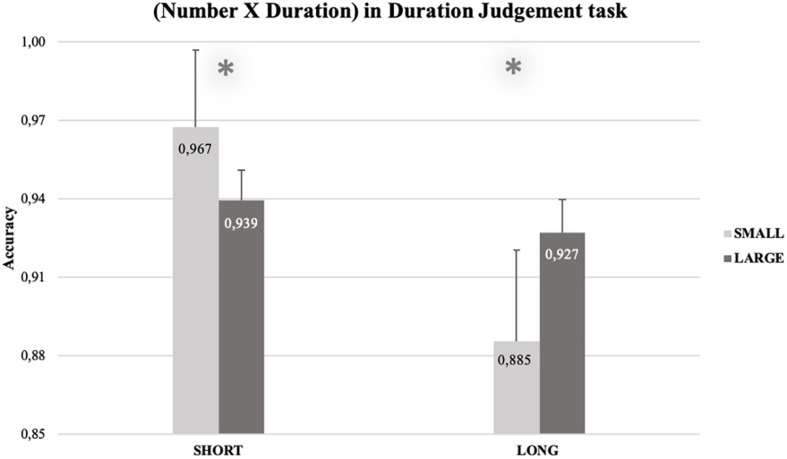
Percentage of correct answers for the duration judgment task, as a function of Duration (short vs. long) and Number (small vs. large). Accuracy for short durations was higher when numbers were small (*M* = 0.967, *SEM* = 0.015) than when they were large (*M* = 0.939, *SEM* = 0.006). Similarly, accuracy for long durations was higher when numbers were large (*M* = 0.927, *SEM* = 0.006) than when they were small (*M* = 0.885, *SEM* = 0.018). The error bars indicate the confidence intervals (95%), corrected for within-subjects designs (O’Brien and Cousineau, 2014; single method).

The RM-ANOVA in the Dual task condition (see Supplementary Table 7) revealed a main effect of Duration, *F*(1, 13) = 9.39, *p* = 0.009, η ^2^*_*p*_* = 0.419. Accuracy was higher with short durations (*M* = 0.953, *SEM* = 0.015) than with long ones (*M* = 0.906, *SEM* = 0.015). The double interaction SOA × Duration was also significant, *F*(1, 13) = 5.05, *p* = 0.043, η ^2^*_*p*_* = 0.280. Paired samples *t*-tests (two-tailed) showed that the simple effect of Duration emerged in both the SOA conditions^4^, even if it was more pronounced, as pointed by the Cohen’s *d*, in the overlap conditions, *t*(13) = 3.53, *p* = 0.004, *MD* = 0.057, *SEM* = 0.016, 95% CI*_*MD*_* [0.022, 0.093], *d* = −0.752, 95% CI*_*d*_* [−1.249, −0.236], than in the no-overlap ones, *t*(13) = 2.34, *p* = 0.036, *MD* = 0.037, *SEM* = 0.016, 95% CI*_*MD*_* [0.003, 0.071], *d* = −0.599, 95% CI*_*d*_* [−1.141, −0.038]. Crucially, the double interaction Number × Duration was significant, *F*(1, 13) = 6.28, *p* = 0.026, η ^2^*_*p*_* = 0.326. Paired-samples *t*-tests (one-tailed) showed a congruency effect for both short {*t*(13) = 2.29, *p* = 0.020, *MD* = 0.028, *SEM* = 0.012, 95% CI*_*MD*_* [0.006, ∞], *d* = −0.563, 95% CI*_*d*_* [−1.080, −0.027]} and long {*t*(13) = 2.40, *p* = 0.016, *MD* = 0.042, *SEM* = 0.017, 95% CI*_*MD*_* [0.011, ∞], *d* = 0.456, 95% CI*_*d*_* [0.037, 0.860]} durations. Accuracy for short durations was higher when paired with small (*M* = 0.967, *SEM* = 0.012) rather than with large (*M* = 0.939, *SEM* = 0.012) numbers. Similarly, accuracy for long durations was higher when paired with large (*M* = 0.927, *SEM* = 0.017), rather than with small (*M* = 0.885, *SEM* = 0.017) numbers (see Figure 4). No other main effects or interactions were significant [all the *F*_*s*_(1, 13) ≤ 1.43].

#### Summary: Accuracy

With reference to our hypotheses, the results on the accuracies in the number comparison task showed an asymmetrical influence of durations on numbers (the congruency effect), indexed by a significant interaction between numbers and durations only for large numbers, and only in the Dual task condition. Thus, only when time is explicitly processed (i.e., in the Dual Task) we found that participants were more accurate when large numbers were paired with long rather than short durations. These results are mirroring, and thus strengthening the findings on the RTs. Furthermore, results on the accuracies in the duration judgment task showed a symmetrical influence of numbers on durations: participants were more accurate when judging long durations paired with large rather than small numbers; similarly, participants were more accurate when judging short durations paired with small rather than large numbers.

## General Discussion

In the following sub-sections we discuss our results, starting from those that were unexpected (no *a priori* hypotheses), and continuing with those which were predicted by our *a priori* hypotheses. The last ones are discussed according to the two theoretical frameworks, ATOM vs. spatial attention orienting (MNL/MTL), described in the section “Introduction.”

### Effects With no *a priori* Hypotheses

#### Effects of SOA, Duration and Their Interaction

We found a main effect of duration and SOA, in both the Single and the Dual Task conditions. The effect of duration has been interpreted as a Foreperiod (FP) effect (Niemi and Näätänen, 1981): participants are faster when they process long rather than short durations. The SOA effect has been interpreted as the result of a greater overlapping between temporal and number processing: participants were slower when stimuli overlapped rather than when they did not overlap.

Moreover, the interaction between SOA (duration-number overlapped) and duration was significant only in the Single Task: the FP effect was greater in the overlap condition. This finding could be interpreted in terms of a slower performance of participants in the overlap condition. Indeed, in this case the temporal stimulus was perceived as a distracter, which, in turn, would have caused an increasing of the positive difference between the RTs for short and long durations. Interestingly, when the cardinal dimension of time was explicitly processed in the Dual Task, the interaction between the FP and the SOA (duration-number overlap) effects disappeared. This finding suggests that the FP effect could be more related to the ordinal dimension of time (i.e., before/after). Indeed, when the temporal duration is processed, the greater salience of the cardinal dimension of time, with respect to the ordinal one, probably canceled out the interaction.

Accuracy results, in the number comparison task, showed a main effect of duration, only in the Dual Task: participants were more accurate when processing long rather than short durations. Taken together, the RT and accuracy findings on the main effect of duration, allow us to rule out the presence of a speed-accuracy trade-off: participants are facilitated (i.e., more accurate and rapid) when processing numbers paired to long durations. The main effect of duration at the level of accuracy, at least when the target stimulus is a number, could be explained in terms of the accuracy counterpart of the FP effect. This is a new finding, because to date the FP effect has been described only in terms of RTs.

### Effects With *a priori* Hypotheses

As stated in the section “Introduction,” the aim of this study was twofold: 1) to investigate the effects of implicit and explicit processing of time on numbers; 2) to investigate the bi-directional interaction between time and numbers.

#### Effects of Implicit and Explicit Processing of Time on Numbers

The answer to our first question comes from the results obtained by analyzing participants’ performance (i.e., RTs and accuracies) in the number comparison task. Globally, RT and accuracy results are coherent and showed that the congruency between numbers and temporal durations emerged only in the Dual Task, and only for large numbers. Indeed, participants were faster and more accurate when processing large numbers paired with long rather than with short durations. Therefore, two different points characterize our expected congruency effect. It emerged only when: (1) time was explicitly processed; (2) participants had to process large numbers (i.e., >5).

The first point suggests that duration needs to be explicitly processed to modulate the representation of numbers. In the domain of number-space interaction, Casarotti et al. (2007) showed that numbers, but not other non-numerical ordinal sequences (i.e., letters of the alphabet), need to be explicitly processed for eliciting an automatic allocation of spatial attention. These findings showed that only the cardinal dimension of numbers (since letters of the alphabet convey ordinal, but no cardinal information), if explicitly processed, could elicit a shift of spatial attention. To the best of our knowledge, to date no study has showed that this is true also for temporal stimuli.

A corpus of empirical evidence, instead, suggests that time is spatially represented (see for review Bonato et al., 2012). Our findings extend the ones of Casarotti et al. (2007) to the domain of number-time interaction: temporal duration modulates number processing only when explicitly processed. According to the spatial attention hypothesis, our interaction between time and numbers should be based on the following mechanism: the explicit classification of duration in terms of short/long enhances the representation of the left/right side of space, which in turn primes the activation of the corresponding left/right spatial locations along the MNL. Importantly, we did not use a spatial response code (i.e., we used a verbal response code: Ti/To or To/Ti counterbalanced across participants), to test whether these interactions would take place at the representational level and not at the level of response selection.

The second point is that the abovementioned time-number interaction emerged only for large numbers. In a first interpretation, this could be explained by the fact that two different effects co-exist when processing variable temporal intervals (i.e., durations): (a) a FP effect, relative to the perception of the durations; (b) a congruency effect, relative to the concurrent activation of a left-to-right spatial layout for representing numbers and durations. These co-existing effects are indexed by opposite patterns of response (in terms of RTs) for small numbers. Indeed, the FP effect (i.e., faster responses for long rather than short durations) is coherent with the number-time congruency effect for large numbers (i.e., faster responses to large numbers paired with long rather than short durations). In contrast, for small numbers the congruency effect (i.e., faster responses for small numbers paired with short rather than long durations) is characterized by a response pattern opposite to that of the FP effect. Therefore, for small numbers, the FP effect could have canceled out the congruency effect.

Alternatively, no congruency effect is present for large numbers, but what we found is just a FP effect for both small and large numbers. Nevertheless, the greater FP effect when processing large rather than small numbers is against this explanation. Furthermore, accuracy findings mirrored the RT ones, and provide strong support to our first interpretation. Participants were more accurate in the congruent rather than in the incongruent conditions, only when time was explicitly processed (i.e., Dual task) and only with large numbers. Indeed, for large numbers, the RT pattern (i.e., the FP effect) was mirrored in the accuracies, allowing us to rule out that this was a case of speed-accuracy tradeoff. For small numbers, however, no significant FP effect emerged for accuracies.

A possible question on our congruency effect can arise: is the congruency effect due to the use of the words “short” and “long” in the duration judgment task, which apply also to spatial extent? Srinivasan and Carey (2010), in their Experiments 4 and 5 on 9-month-old infants, have shown that this was not the case. They have suggested, instead, that it may reflect an evolutionary recycling of spatial representations for more general purposes.

#### Reciprocal Interaction Between Time and Numbers

Our second question has been answered by the accuracy results in the Dual Task condition, which have shown the existence of an asymmetric relation between numbers and time. Accuracy results in the duration judgment task showed a congruency effect for both small and large numbers: participants were more accurate when judging long durations after having processed large rather than small numbers; analogously, participants were more accurate when judging short durations after having processed small rather than large numbers. In the number comparison task, however, accuracy results just showed that the hypothesized congruency effect emerged only for large numbers (as previously described).

We can interpret these findings, according to the view that numbers influence time more than vice-versa, at least at the level of accuracy. These findings are not in line with the ATOM theory. Indeed, if a common system for quantity representation do exist, we should have observed a symmetric interaction between the number and the temporal domain. Instead, the presence of an asymmetric effect suggests that the representations of numbers and time are, at least, partially independent. This is in line with the hypothesis on the existence of a MNL and a MTL based on the mechanism of spatial attention orienting. Indeed, on one hand, number processing could have produced an automatic shift of spatial attention, which in turn enhanced a specific side of the physical space (i.e., the left side with small numbers and the right side with large numbers). The activation of the physical space, in turn, might have enhanced the corresponding side of the temporal space (i.e., short durations on the left side and long durations on the right side). On the other hand, only the explicit processing of long durations could have produced a shift of spatial attention, enhancing the right side of the physical space, which, in turn, might have enhanced the corresponding right side of the number space. Moreover, we speculate that a hierarchical representation for numbers and time could exist when both distinct numerical and temporal stimuli are explicitly processed. Indeed, studies on number-space interactions suggest that spatial attention modulates numbers independently from whether numbers are implicitly or explicitly processed (see Dehaene et al., 1993). In contrast, numbers modulate the allocation of spatial attention only when numbers are explicitly processed (Casarotti et al., 2007). These findings index a sort of hierarchical representation between space and numbers, where space influences numbers more than vice-versa. Our findings extend the ones of Casarotti et al. (2007) to the level of number-time interaction: temporal duration modulates number processing only when explicitly processed, whereas the influence of numbers on time seems to be more automatic.

## Conclusion

Whether time and numbers share a common spatial representation or independent representations exist, and can interact in a task-dependent way, is still an open and intriguing issue. First, our results showed the presence of two simultaneous effects for time (i.e., the FP and the congruency effect) that could reflect the existence of concurrent but different processes for the estimation and the classification of durations, for which different representations should be needed. In this case, a spatial representation of time could be involved only when required by the specific task, such as duration comparison (Di Bono et al., 2012) or classification (the present study). Crucially, we found that time should be explicitly processed for influencing number processing. Moreover, when both numbers and time are explicitly processed, numbers influence time more than vice-versa.

These findings suggest that the representations of numbers and time are at least partially independent. Moreover, the fact that also spatial attention influences numbers more than vice-versa (e.g., Casarotti et al., 2007) suggests that number-space and number-time interactions could have a hierarchical nature, on the basis of a common property: the level of abstraction. Indeed, with reference to space-number/time interactions, it has been hypothesized that space is used to reason about more abstract concepts, such as symbolic numbers and time (Bonato and Umiltà, 2014). Whether and how we use both space and number-space representations, for coding time, depending on the specific task (e.g., estimation vs. classification), remains an intriguing question.

## Data Availability Statement

All datasets generated for this study are included in the article/Supplementary Material.

## Ethics Statement

The studies involving human participants were reviewed and approved by the Institutional Review Board at the University of Padua, Italy. The patients/participants provided their written informed consent to participate in this study.

## Author Contributions

MD, SC, and KP contributed to the conception and design of the study. CD collected the data and contributed in writing the first draft of the manuscript. MD and CD analyzed the data. MD wrote the first draft of the manuscript. MD, CD, SC, and KP interpreted the results, read and contributed to the final version of the manuscript.

## Conflict of Interest

The authors declare that the research was conducted in the absence of any commercial or financial relationships that could be construed as a potential conflict of interest.

## Footnotes

1 Mean RTs in no-overlap condition (Single Task: *M* = 528.70 ms, *SEM* = 2.514; Dual Task: *M* = 838.82 ms, *SEM* = 3.808) and overlap condition (Single Task: *M* = 554.51 ms, *SEM* = 2.514; Dual Task: *M* = 891.06 ms, *SEM* = 3.808).

2 Short durations (non-overlap: *M* = 699.21 ms, *SEM* = 3.173; overlap: *M* = 745.21 ms, *SEM* = 3.173); long durations (no-overlap: *M* = 657.62 ms, *SEM* = 4.929; overlap: *M* = 689.53 ms, *SEM* = 4.929).

3 No-overlap (short: *M* = 699.21 ms, *SEM* = 5.149; long: *M* = 657.62 ms, *SEM* = 5.149); overlap (short: *M* = 744.44 ms, *SEM* = 5.127; long: *M* = 689.53 ms, *SEM* = 5.127).

4 Mean accuracy in no-overlap (short: *M* = 0.952, *SEM* = 0.016; long: *M* = 0.915, *SEM* = 0.016) and overlap (short: *M* = 0.955, *SEM* = 0.016; long: *M* = 0.898, *SEM* = 0.016) conditions.
