# Supplementary material for: Can Implicit or Explicit Time Processing Impact Numerical Representation? Evidence From a Dual Task Paradigm
Source: Front Psychol. 2020 Jan 8;10:2882. doi: 10.3389/fpsyg.2019.02882 (PMC6960196; doi:10.3389/fpsyg.2019.02882)
Supplement: Supplementary file 3 [file Table_1.DOCX]

Supplementary Material

| **Table 1. Results of the mixed ANOVA performed on the mean RTs in number comparison.** | | | | | | | | | | | | | | | | | | | | |
| --- | --- | --- | --- | --- | --- | --- | --- | --- | --- | --- | --- | --- | --- | --- | --- | --- | --- | --- | --- | --- |
|  | | | | | | | **Sum of Squares** | | **df** | | **Mean Square** | | | | **F** | | **p** | | **η² _p_** | |
| **SOA** | | | | | |  | 86276.1 |  | 1 |  | 86276.1 | | |  | 293.589 |  | **< .001** |  | 0.916 |  |
| **SOA ✻ TASK** | | | | | |  | 10124.1 |  | 1 |  | 10124.1 | | |  | 34.451 |  | **< .001** |  | 0.561 |  |
| Residual | | | | | |  | 7934.4 |  | 27 |  | 293.9 | | |  |  |  |  |  |  |  |
| NUMBER | | | | | |  | 1940.1 |  | 1 |  | 1940.1 | | |  | 0.581 |  | 0.453 |  | 0.021 |  |
| NUMBER ✻ TASK | | | | | |  | 330.5 |  | 1 |  | 330.5 | | |  | 0.099 |  | 0.755 |  | 0.004 |  |
| Residual | | | | | |  | 90162.4 |  | 27 |  | 3339.3 | | |  |  |  |  |  |  |  |
| **DURATION** | | | | | |  | 135008.2 |  | 1 |  | 135008.2 | | |  | 120.301 |  | **< .001** |  | 0.817 |  |
| DURATION ✻ TASK | | | | | |  | 2733.5 |  | 1 |  | 2733.5 | | |  | 2.436 |  | 0.130 |  | 0.083 |  |
| Residual | | | | | |  | 30300.9 |  | 27 |  | 1122.3 | | |  |  |  |  |  |  |  |
| SOA ✻ NUMBER | | | | | |  | 465.5 |  | 1 |  | 465.5 | | |  | 0.569 |  | 0.457 |  | 0.021 |  |
| SOA ✻ NUMBER ✻ TASK | | | | | |  | 475.3 |  | 1 |  | 475.3 | | |  | 0.581 |  | 0.452 |  | 0.021 |  |
| Residual | | | | | |  | 22079.8 |  | 27 |  | 817.8 | | |  |  |  |  |  |  |  |
| **SOA ✻ DURATION** | | | | | |  | 2570.5 |  | 1 |  | 2570.5 | | |  | 8.503 |  | **0.007** |  | 0.240 |  |
| **SOA ✻ DURATION ✻ TASK** | | | | | |  | 1676.7 |  | 1 |  | 1676.7 | | |  | 5.546 |  | **0.026** |  | 0.170 |  |
| Residual | | | | | |  | 8162.0 |  | 27 |  | 302.3 | | |  |  |  |  |  |  |  |
| **NUMBER ✻ DURATION** | | | | | |  | 3801.8 |  | 1 |  | 3801.8 | | |  | 4.815 |  | **0.037** |  | 0.151 |  |
| **NUMBER ✻ DURATION ✻ TASK** | | | | | |  | 2890.9 |  | 1 |  | 2890.9 | | |  | 3.662 |  | **0.066** |  | 0.119 |  |
| Residual | | | | | |  | 21317.7 |  | 27 |  | 789.5 | | |  |  |  |  |  |  |  |
| SOA ✻ NUMBER ✻ DURATION | | | | | |  | 117.4 |  | 1 |  | 117.4 | | |  | 0.456 |  | 0.505 |  | 0.017 |  |
| SOA ✻ NUMBER ✻ DURATION ✻ TASK | | | | | |  | 663.8 |  | 1 |  | 663.8 | | |  | 2.576 |  | 0.120 |  | 0.087 |  |
| Residual | | | | | |  | 6957.7 |  | 27 |  | 257.7 | | |  |  |  |  |  |  |  |
|  | | | | | | | | | | | | | | | | | | | | |
|  | | | | | | | | | | | | | | | | | | | | |
| **Between Subjects Effects** | | | | | | | | | | | | | |  |  |  |  |  |  |  |
|  | | **Sum of Squares** | | **df** | | **Mean Square** | | **F** | | **p** | | **η²** | |  |  |  |  |  |  |  |
| **TASK** |  | 6.057e +6 |  | 1 |  | 6.057e +6 |  | 51.20 |  | **< .001** |  | 0.655 |  |  |  |  |  |  |  |  |
| Residual |  | 3.194e +6 |  | 27 |  | 118290 |  |  |  |  |  |  |  |  |  |  |  |  |  |  |
|  | | | | | | | | | | | | | |  |  |  |  |  |  |  |
|  | | | | | | | | | | | | | |  |  |  |  |  |  |  |

| **Table 2. Results of the RM-ANOVA performed on the mean RTs in number comparison (Single Task condition - implicit elaboration of time).** | | | | | | | | | | | | | |
| --- | --- | --- | --- | --- | --- | --- | --- | --- | --- | --- | --- | --- | --- |
|  | | **Sum of Squares** | | **df** | | **Mean Square** | | **F** | | **p** | | **η² _p_** | |
| **SOA** |  | 19976.115 |  | 1 |  | 19976.115 |  | 105.340 |  | **< .001** |  | 0.883 |  |
| Residual |  | 2654.889 |  | 14 |  | 189.635 |  |  |  |  |  |  |  |
| **DURATION** |  | 51952.249 |  | 1 |  | 51952.249 |  | 57.233 |  | **< .001** |  | 0.803 |  |
| Residual |  | 12708.331 |  | 14 |  | 907.738 |  |  |  |  |  |  |  |
| NUMBER |  | 362.783 |  | 1 |  | 362.783 |  | 0.265 |  | 0.614 |  | 0.019 |  |
| Residual |  | 19130.398 |  | 14 |  | 1366.457 |  |  |  |  |  |  |  |
| **SOA ✻ DURATION** |  | 4213.765 |  | 1 |  | 4213.765 |  | 22.994 |  | **< .001** |  | 0.622 |  |
| Residual |  | 2565.597 |  | 14 |  | 183.257 |  |  |  |  |  |  |  |
| SOA ✻ NUMBER |  | 0.136 |  | 1 |  | 0.136 |  | 6.427e -4 |  | 0.980 |  | 0.000 |  |
| Residual |  | 2957.124 |  | 14 |  | 211.223 |  |  |  |  |  |  |  |
| DURATION ✻ NUMBER |  | 48.810 |  | 1 |  | 48.810 |  | 0.349 |  | 0.564 |  | 0.024 |  |
| Residual |  | 1957.387 |  | 14 |  | 139.813 |  |  |  |  |  |  |  |
| SOA ✻ DURATION ✻ NUMBER |  | 102.150 |  | 1 |  | 102.150 |  | 0.518 |  | 0.483 |  | 0.036 |  |
| Residual |  | 2758.904 |  | 14 |  | 197.065 |  |  |  |  |  |  |  |
|  | | | | | | | | | | | | | |
|  | | | | | | | | | | | | | |

| **Table 3. Results of the RM-ANOVA performed on the mean RTs in number comparison (Dual Task condition - explicit elaboration of time).** | | | | | | | | | | | | | | | | | | | | | | |  |  |
| --- | --- | --- | --- | --- | --- | --- | --- | --- | --- | --- | --- | --- | --- | --- | --- | --- | --- | --- | --- | --- | --- | --- | --- | --- |
|  | | | | | | | **Sum of Squares** | | | | | | **df** | | **Mean Square** | | **F** | | **p** | | **η² _p_** | |  |  |
| **SOA** | | | | | |  | 76424.14 | | | |  | | 1 |  | 76424.14 |  | 188.182 |  | **< .001** |  | 0.935 |  |  |  |
| Residual | | | | | |  | 5279.53 | | | |  | | 13 |  | 406.12 |  |  |  |  |  |  |  |  |  |
| NUMBER | | | | | |  | 1907.80 | | | |  | | 1 |  | 1907.80 |  | 0.349 |  | 0.565 |  | 0.026 |  |  |  |
| Residual | | | | | |  | 71031.99 | | | |  | | 13 |  | 5464.00 |  |  |  |  |  |  |  |  |  |
| **DURATION** | | | | | |  | 85789.46 | | | |  | | 1 |  | 85789.46 |  | 63.394 |  | **< .001** |  | 0.830 |  |  |  |
| Residual | | | | | |  | 17592.59 | | | |  | | 13 |  | 1353.28 |  |  |  |  |  |  |  |  |  |
| SOA ✻ NUMBER | | | | | |  | 940.64 | | | |  | | 1 |  | 940.64 |  | 0.639 |  | 0.438 |  | 0.047 |  |  |  |
| Residual | | | | | |  | 19122.65 | | | |  | | 13 |  | 1470.97 |  |  |  |  |  |  |  |  |  |
| SOA ✻ DURATION | | | | | |  | 33.38 | | | |  | | 1 |  | 33.38 |  | 0.078 |  | 0.785 |  | 0.006 |  |  |  |
| Residual | | | | | |  | 5596.36 | | | |  | | 13 |  | 430.49 |  |  |  |  |  |  |  |  |  |
| **NUMBER ✻ DURATION** | | | | | |  | 6643.88 | | | |  | | 1 |  | 6643.88 |  | 4.461 |  | **0.055** |  | 0.255 |  |  |  |
| Residual | | | | | |  | 19360.29 | | | |  | | 13 |  | 1489.25 |  |  |  |  |  |  |  |  |  |
| SOA ✻ NUMBER ✻ DURATION | | | | | |  | 679.07 | | | |  | | 1 |  | 679.07 |  | 2.102 |  | 0.171 |  | 0.139 |  |  |  |
| Residual | | | | | |  | 4198.80 | | | |  | | 13 |  | 322.98 |  |  |  |  |  |  |  |  |  |
|  | | | | | | | | | | | | | | | | | | | | | | |  |  |
|  | | | | | | | | | | | | | | | | | | | | | | |  |  |
| **Paired Samples T-Test: NUMBER x DURATION** | | | | | | | | | | | | | | | | | | | | | | | |  |
|  | | | | | | | | | | | | | | | | | | | | **95% Confidence interval** | | | |  |
|  | |  | |  | | **t** | | **df** | | **p** | **Mean Difference** | | | | | **SE Difference** | | **Cohen's d** | | **Lower** | | **Upper** | |  |
| SMALL-SHORT |  | - |  | SMALL-LONG |  | 4.732 |  | 13 |  | 1.000 |  | 39.95 | | |  | 8.442 |  | 1.265 |  | -∞ |  | 54.90 |  | |
| LARGE-LONG |  | - |  | LARGE-SHORT |  | -6.164 |  | 13 |  | **< .001** |  | -70.76 | | |  | 11.479 |  | -1.647 |  | -∞ |  | -50.43 |  | |
|  | | | | | | | | | | | | | | | | | | | | | | | |  |

| **Table 4. Results of the mixed ANOVA performed on the mean Accuracy in number comparison.** | | | | | | | | | | | | | | | | |  |
| --- | --- | --- | --- | --- | --- | --- | --- | --- | --- | --- | --- | --- | --- | --- | --- | --- | --- |
|  | | | **Sum of Squares** | | | | **df** | | **Mean Square** | | **F** | | **p** | | **η² _p_** | |  |
| SOA | |  | 9.273e -5 | | |  | 1 |  | 9.273e -5 |  | 0.292 |  | 0.594 |  | 0.011 |  |  |
| **SOA ✻ TASK** | |  | 0.001 | | |  | 1 |  | 0.001 |  | 4.498 |  | **0.043** |  | 0.143 |  |  |
| Residual | |  | 0.009 | | |  | 27 |  | 3.180e -4 |  |  |  |  |  |  |  |  |
| NUMBER | |  | 0.001 | | |  | 1 |  | 0.001 |  | 3.380 |  | 0.077 |  | 0.111 |  |  |
| NUMBER ✻ TASK | |  | 1.465e -5 | | |  | 1 |  | 1.465e -5 |  | 0.048 |  | 0.829 |  | 0.002 |  |  |
| Residual | |  | 0.008 | | |  | 27 |  | 3.071e -4 |  |  |  |  |  |  |  |  |
| **DURATION** | |  | 0.001 | | |  | 1 |  | 0.001 |  | 10.085 |  | **0.004** |  | 0.272 |  |  |
| **DURATION ✻ TASK** | |  | 6.378e -4 | | |  | 1 |  | 6.378e -4 |  | 4.956 |  | **0.035** |  | 0.155 |  |  |
| Residual | |  | 0.003 | | |  | 27 |  | 1.287e -4 |  |  |  |  |  |  |  |  |
| SOA ✻ NUMBER | |  | 1.157e -4 | | |  | 1 |  | 1.157e -4 |  | 0.471 |  | 0.498 |  | 0.017 |  |  |
| SOA ✻ NUMBER ✻ TASK | |  | 1.179e -4 | | |  | 1 |  | 1.179e -4 |  | 0.480 |  | 0.494 |  | 0.017 |  |  |
| Residual | |  | 0.007 | | |  | 27 |  | 2.455e -4 |  |  |  |  |  |  |  |  |
| SOA ✻ DURATION | |  | 7.413e -5 | | |  | 1 |  | 7.413e -5 |  | 0.271 |  | 0.607 |  | 0.010 |  |  |
| SOA ✻ DURATION ✻ TASK | |  | 2.796e -5 | | |  | 1 |  | 2.796e -5 |  | 0.102 |  | 0.752 |  | 0.004 |  |  |
| Residual | |  | 0.007 | | |  | 27 |  | 2.737e -4 |  |  |  |  |  |  |  |  |
| **NUMBER ✻ DURATION** | |  | 0.003 | | |  | 1 |  | 0.003 |  | 4.867 |  | **0.036** |  | 0.153 |  |  |
| **NUMBER ✻ DURATION ✻ TASK** | |  | 0.003 | | |  | 1 |  | 0.003 |  | 4.240 |  | **0.049** |  | 0.136 |  |  |
| Residual | |  | 0.016 | | |  | 27 |  | 6.079e -4 |  |  |  |  |  |  |  |  |
| SOA ✻ NUMBER ✻ DURATION | |  | 7.434e -5 | | |  | 1 |  | 7.434e -5 |  | 0.644 |  | 0.429 |  | 0.023 |  |  |
| SOA ✻ NUMBER ✻ DURATION ✻ TASK | |  | 3.055e -6 | | |  | 1 |  | 3.055e -6 |  | 0.026 |  | 0.872 |  | 0.001 |  |  |
| Residual | |  | 0.003 | | |  | 27 |  | 1.154e -4 |  |  |  |  |  |  |  |  |
|  | | | | | | | | | | | | | | | | |  |
|  | | | | | | | | | | | | | | | | |  |
| **Between Subjects Effects** | | | | | | | | | | | | | | | | | |
|  | | **Sum of Squares** | | **df** | | **Mean Square** | | | | **F** | | **p** | | **η²** | | | |
| TASK. Single = 1 |  | 0.011 |  | 1 |  | 0.011 | | |  | 2.582 |  | 0.120 |  | 0.087 | | |  |
| Residual |  | 0.116 |  | 27 |  | 0.004 | | |  |  |  |  |  |  | | |  |
|  | | | | | | | | | | | | | | | | | |
|  | | | | | | | | | | | | | | | | | |

| **Table 5. Results of the RM-ANOVA performed on the mean Accuracy in number comparison (Single Task condition - implicit elaboration of time).** | | | | | | | | | | | | | |
| --- | --- | --- | --- | --- | --- | --- | --- | --- | --- | --- | --- | --- | --- |
|  | | **Sum of Squares** | | **df** | | **Mean Square** | | **F** | | **p** | | **η²** | |
| SOA |  | 4.116e -4 |  | 1 |  | 4.116e -4 |  | 1.602 |  | 0.226 |  | 0.103 |  |
| Residual |  | 0.004 |  | 14 |  | 2.569e -4 |  |  |  |  |  |  |  |
| NUMBER |  | 4.174e -4 |  | 1 |  | 4.174e -4 |  | 2.217 |  | 0.159 |  | 0.137 |  |
| Residual |  | 0.003 |  | 14 |  | 1.883e -4 |  |  |  |  |  |  |  |
| DURATION |  | 6.008e -5 |  | 1 |  | 6.008e -5 |  | 0.791 |  | 0.389 |  | 0.053 |  |
| Residual |  | 0.001 |  | 14 |  | 7.598e -5 |  |  |  |  |  |  |  |
| SOA ✻ NUMBER |  | 5.252e -9 |  | 1 |  | 5.252e -9 |  | 2.572e -5 |  | 0.996 |  | 0.000 |  |
| Residual |  | 0.003 |  | 14 |  | 2.042e -4 |  |  |  |  |  |  |  |
| SOA ✻ DURATION |  | 5.716e -6 |  | 1 |  | 5.716e -6 |  | 0.038 |  | 0.847 |  | 0.003 |  |
| Residual |  | 0.002 |  | 14 |  | 1.485e -4 |  |  |  |  |  |  |  |
| NUMBER ✻ DURATION |  | 6.803e -6 |  | 1 |  | 6.803e -6 |  | 0.021 |  | 0.888 |  | 0.001 |  |
| Residual |  | 0.005 |  | 14 |  | 3.281e -4 |  |  |  |  |  |  |  |
| SOA ✻ NUMBER ✻ DURATION |  | 5.569e -5 |  | 1 |  | 5.569e -5 |  | 0.540 |  | 0.475 |  | 0.037 |  |
| Residual |  | 0.001 |  | 14 |  | 1.032e -4 |  |  |  |  |  |  |  |
|  | | | | | | | | | | | | | |
|  | | | | | | | | | | | | | |

| **Table 6. Results of the RM-ANOVA performed on the mean Accuracy in number comparison (Dual Task condition - explicit elaboration of time).** | | | | | | | | | | | | | | | | | | | | | | | | | | | | | | | | | | | |
| --- | --- | --- | --- | --- | --- | --- | --- | --- | --- | --- | --- | --- | --- | --- | --- | --- | --- | --- | --- | --- | --- | --- | --- | --- | --- | --- | --- | --- | --- | --- | --- | --- | --- | --- | --- |
|  | | | | | | | | | | | | | **Sum of Squares** | | | | **df** | | | | | | **Mean Square** | | | | **F** | | | | **p** | | | **η²** | |
| SOA | | | | | | |  | | | | | | 0.001 | | |  | | 1 | | | |  | 0.001 | | |  | 2.836 | | |  | 0.116 | |  | 0.179 |  |
| Residual | | | | | | |  | | | | | | 0.005 | | |  | | 13 | | | |  | 3.837e -4 | | |  |  | | |  |  | |  |  |  |
| NUMBER | | | | | | |  | | | | | | 6.280e -4 | | |  | | 1 | | | |  | 6.280e -4 | | |  | 1.443 | | |  | 0.251 | |  | 0.100 |  |
| Residual | | | | | | |  | | | | | | 0.006 | | |  | | 13 | | | |  | 4.351e -4 | | |  |  | | |  |  | |  |  |  |
| **DURATION** | | | | | | |  | | | | | | 0.002 | | |  | | 1 | | | |  | 0.002 | | |  | 9.787 | | |  | **0.008** | |  | 0.429 |  |
| Residual | | | | | | |  | | | | | | 0.002 | | |  | | 13 | | | |  | 1.855e -4 | | |  |  | | |  |  | |  |  |  |
| SOA ✻ NUMBER | | | | | | |  | | | | | | 2.258e -4 | | |  | | 1 | | | |  | 2.258e -4 | | |  | 0.779 | | |  | 0.394 | |  | 0.057 |  |
| Residual | | | | | | |  | | | | | | 0.004 | | |  | | 13 | | | |  | 2.900e -4 | | |  |  | | |  |  | |  |  |  |
| SOA ✻ DURATION | | | | | | |  | | | | | | 9.335e -5 | | |  | | 1 | | | |  | 9.335e -5 | | |  | 0.228 | | |  | 0.641 | |  | 0.017 |  |
| Residual | | | | | | |  | | | | | | 0.005 | | |  | | 13 | | | |  | 4.086e -4 | | |  |  | | |  |  | |  |  |  |
| **NUMBER ✻ DURATION** | | | | | | |  | | | | | | 0.005 | | |  | | 1 | | | |  | 0.005 | | |  | 5.879 | | |  | **0.031** | |  | 0.311 |  |
| Residual | | | | | | |  | | | | | | 0.012 | | |  | | 13 | | | |  | 9.093e -4 | | |  |  | | |  |  | |  |  |  |
| SOA ✻ NUMBER ✻ DURATION | | | | | | |  | | | | | | 2.284e -5 | | |  | | 1 | | | |  | 2.284e -5 | | |  | 0.178 | | |  | 0.680 | |  | 0.013 |  |
| Residual | | | | | | |  | | | | | | 0.002 | | |  | | 13 | | | |  | 1.286e -4 | | |  |  | | |  |  | |  |  |  |
|  | | | | | | | | | | | | | | | | | | | | | | | | | | | | | | | | | | | |
|  | | | | | | | | | | | | | | | | | | | | | | | | | | | | | | | | | | | |
| **Paired Samples T-Test: NUMBER x DURATION** | | | | | | | | | | | | | | | | | | | | | | | | | | | | | | | | | | | |
|  | | | | | | | | | | | | | | | | | | | | | | | | | | | | | **95% Confidence Interval** | | | | | | |
|  | |  | |  | | **t** | | | **df** | | | **p** | | | **Mean Difference** | | | | **SE Difference** | | | | | | **Cohen's d** | | | | **Lower** | | | | **Upper** | | |
| SMALL-SHORT |  | - |  | SMALL-LONG |  | 1.23 | |  | | 13 |  | 0.12 | |  | 0.006 | | | | |  | 0.005 | | |  | 0.327 | | |  | -0.003 | | |  | ∞ | |  |
| LARGE  -LONG |  | - |  | LARGE-SHORT |  | 2.92 | |  | 13 | |  | **0.006** | |  | 0.022 | | | | |  | 0.007 | | |  | 0.781 | | |  | 0.009 | | |  | ∞ | |  |
|  | | | | | | | | | | | | | | | | | | | | | | | | | | | | | | | | | | | |
|  | | | | | | | | | | | | | | | | | | | | | | | | | | | | | | | | | | | |

| **Table 7. Results of the RM-ANOVA performed on the mean Accuracy in duration judgement (Dual Task condition - explicit elaboration of time).** | | | | | | | | | | | | | | | | | | | | | | |  |  |  |
| --- | --- | --- | --- | --- | --- | --- | --- | --- | --- | --- | --- | --- | --- | --- | --- | --- | --- | --- | --- | --- | --- | --- | --- | --- | --- |
|  | | | | | | | **Sum of Squares** | | | | | | **df** | | **Mean Square** | | **F** | | **p** | | **η² _p_** | |  |  |  |
| SOA | | | | | |  | 0.001 | | | | |  | 1 |  | 0.001 |  | 1.010 |  | 0.333 |  | 0.072 |  |  |  |  |
| Residual | | | | | |  | 0.017 | | | | |  | 13 |  | 0.001 |  |  |  |  |  |  |  |  |  |  |
| NUMBER | | | | | |  | 0.001 | | | | |  | 1 |  | 0.001 |  | 1.426 |  | 0.254 |  | 0.099 |  |  |  |  |
| Residual | | | | | |  | 0.012 | | | | |  | 13 |  | 9.058e -4 |  |  |  |  |  |  |  |  |  |  |
| **DURATION** | | | | | |  | 0.063 | | | | |  | 1 |  | 0.063 |  | 9.392 |  | **0.009** |  | 0.419 |  |  |  |  |
| Residual | | | | | |  | 0.087 | | | | |  | 13 |  | 0.007 |  |  |  |  |  |  |  |  |  |  |
| SOA ✻ NUMBER | | | | | |  | 1.765e -4 | | | | |  | 1 |  | 1.765e -4 |  | 0.138 |  | 0.716 |  | 0.011 |  |  |  |  |
| Residual | | | | | |  | 0.017 | | | | |  | 13 |  | 0.001 |  |  |  |  |  |  |  |  |  |  |
| **SOA ✻ DURATION** | | | | | |  | 0.003 | | | | |  | 1 |  | 0.003 |  | 5.055 |  | **0.043** |  | 0.280 |  |  |  |  |
| Residual | | | | | |  | 0.007 | | | | |  | 13 |  | 5.734e -4 |  |  |  |  |  |  |  |  |  |  |
| **NUMBER ✻ DURATION** | | | | | |  | 0.034 | | | | |  | 1 |  | 0.034 |  | 6.280 |  | **0.026** |  | 0.326 |  |  |  |  |
| Residual | | | | | |  | 0.070 | | | | |  | 13 |  | 0.005 |  |  |  |  |  |  |  |  |  |  |
| SOA ✻ NUMBER ✻ DURATION | | | | | |  | 2.845e -4 | | | | |  | 1 |  | 2.845e -4 |  | 0.352 |  | 0.563 |  | 0.026 |  |  |  |  |
| Residual | | | | | |  | 0.011 | | | | |  | 13 |  | 8.077e -4 |  |  |  |  |  |  |  |  |  |  |
|  | | | | | | | | | | | | | | | | | | | | | | |  |  |  |
|  | | | | | | | | | | | | | | | | | | | | | | |  |  |  |
| **Paired Samples T-Test: NUMBER x DURATION** | | | | | | | | | | | | | | | | | | | | | | | | | |
|  | | | | | | | | | | | | | | | | | | | | | | **95% Confidence Interval** | | | |
|  | |  | |  | | **t** | | **df** | | **p** | | **Mean Difference** | | | | **SE Difference** | | **Cohen's d** | | | | **Lower** | | **Upper** | |
| SMALL-SHORT |  | - |  | LARGE-SHORT |  | 2.293 |  | 13 |  | **0.020** |  | 0.028 | | |  | 0.012 |  | 0.613 | | |  | 0.006 |  | ∞ |  |
| SMALL-LONG |  | - |  | LARGE-LONG |  | 2.396 |  | 13 |  | **0.016** |  | 0.042 | | |  | 0.017 |  | -0.640 | | |  | 0.011 |  | ∞ |  |
|  | | | | | | | | | | | | | | | | | | | | | | | | | |
|  | | | | | | | | | | | | | | | | | | | | | | | | | |
